# “Part of the solution yet part of the problem” Stigmatization in mental health professionals: characteristics and associated factors

**DOI:** 10.1192/j.eurpsy.2022.542

**Published:** 2022-09-01

**Authors:** K.-M. Valery, A. Prouteau, L. Violeau, T. Fournier, S. Guionnet

**Affiliations:** University of Bordeaux, Psychology, Bordeaux, France

**Keywords:** stigmatization, schizophrénia, mental health professionals

## Abstract

**Introduction:**

The consequences of schizophrenia stigma are numerous and highly damaging to individuals, their families, the health care system and society. Mental health professionals (MHP) are considered to be one of the main sources of schizophrenia stigmatization.

**Objectives:**

The aim of the study was to identify individual and contextual factors associated with stigmatization in MHP in its three dimensions.

**Methods:**

An online survey was conducted with specific measures of MHP stigmatization (stereotypes, prejudices and discrimination). Four categories of potential associated factors were also measured: sociodemographic information, contextual characteristics (e.g. work setting), individual characteristics (e.g. profession, recovery-oriented practices) and theoretical beliefs (e.g. biological beliefs, perceived similarities, continuum beliefs). Models of prediction were computed when applicable.

**Results:**

Responses of 357 MHP were analysed. The main factors associated with stigmatization (stereotypes, prejudice) in MHP are of two types: i) individual beliefs (about mental illness: biological etiological beliefs, categorical beliefs; or about MHP themselves: professional utility beliefs, similarity beliefs) and ii) characteristics of practices (recovery oriented practice, work setting, profession).

**Conclusions:**

These original results suggest new strategies for reducing stigma in mental health practices such as focusing on individual beliefs and fostering recovery-oriented practice and professional utility beliefs.

**Disclosure:**

No significant relationships.

